# Nutritional Composition, Phenolic Profile, Antioxidant, and Antimicrobial Activity of Bilberry, Lingonberry, and Blueberry Cultivars

**DOI:** 10.1155/ijfo/4181376

**Published:** 2026-06-13

**Authors:** Emmanuel Duah Osei, Anthony Amotoe-Bondzie, Alfred Elikem Kwami Afedzi, Eva Ivanišová, Ľuboš Harangozo, Július Árvay, Dominika Ďurkáčová, Christian R. Encina-Zelada, Branislav Gálik, Miroslava Kačániová, Newlove Akowuah Afoakwah

**Affiliations:** ^1^ School of Food Science and Environmental Health, Technological University Dublin, Dublin, Ireland; ^2^ Sustainability and Health Research Hub, Technological University Dublin, Dublin, Ireland; ^3^ Institute of Food Sciences, Faculty of Biotechnology and Food Sciences, Slovak University of Agriculture in Nitra, Nitra, Slovakia, uniag.sk; ^4^ Lumpkin College of Business and Technology, School of Technology, Eastern Illinois University, Charleston, Illinois, USA, eiu.edu; ^5^ Department of Biotechnology, Fermentation Technology Research Centre, Faculty of Agro-Industry, Kasetsart University, Bangkok, Thailand, ku.ac.th; ^6^ Food Incubator, AgroBioTech Research Centrum, Slovak University of Agriculture in Nitra, Nitra, Slovakia, uniag.sk; ^7^ Department of Food Technology, Faculty of Food Industries, Universidad Nacional Agraria La Molina, Lima, Peru, lamolina.edu.pe; ^8^ Instituto de Investigación de Bioquímica y Biología Molecular, Universidad Nacional Agraria La Molina, Lima, Peru, lamolina.edu.pe; ^9^ Institute of Nutrition and Genomics, Faculty of Agrobiology and Food Resources, Slovak University of Agriculture in Nitra, Nitra, Slovakia, uniag.sk; ^10^ Institute of Horticulture, Faculty of Horticulture and Landscape Engineering, Slovak University of Agriculture in Nitra, Nitra, Slovakia, uniag.sk; ^11^ School of Medical and Health Sciences, University of Economics and Human Sciences in Warsaw, Warsaw, Poland; ^12^ Department of Food Science and Technology, Faculty of Agriculture, Food and Consumer Sciences, Nyankpala Campus, University for Development Studies, Tamale, Ghana, uds.edu.gh

**Keywords:** antimicrobial activity, antioxidants, berries, fatty acids, minerals, phenolic profiles

## Abstract

Berries of the *Vaccinium* genus are valued for their nutritional and bioactive potential; however, differences among species and cultivars remain insufficiently characterized. This study compared the proximate composition, phenolic profiles, fatty acids, mineral content, antioxidant capacity, and antimicrobial activity of bilberry (*Vaccinium myrtillus* “Natural”), lingonberry (*Vaccinium vitis-idaea* “Linnea” and “Sanna”), and highbush blueberry (*Vaccinium corymbosum* “Spartan,” “Bluecrop,” and “Chandler”). Bilberry showed the highest total phenolic content, anthocyanin concentration, antioxidant activity, polyunsaturated fatty acids (PUFAs), and antifungal activity. Lingonberries combined moderate‐to‐high antioxidant capacity with elevated mineral levels, favorable n6/n3 ratios, and potent inhibition of *Candida* species. Blueberries were rich in monounsaturated fatty acids (MUFAs) and exhibited distinct phenolic acid profiles. Correlation analysis revealed strong associations between phenolic content and antioxidant activity, as well as antifungal activity. Principal component analysis distinguished bilberry for its phenolic–PUFA dominance, lingonberry for its phenolic–mineral balance, and blueberries for their MUFA‐rich profiles. These findings demonstrate that cultivar‐specific biochemical traits underpin functional differences, presenting bilberries and lingonberries as promising candidates for sustainable and health‐focused foods and blueberries as a potential option enriched in MUFA for targeted health applications.

## 1. Introduction

Across global food systems, berries of the *Vaccinium* genus, including blueberries (*Vaccinium corymbosum* L.), lingonberries (*Vaccinium vitis-idaea* L.), and bilberries (*Vaccinium myrtillus* L.), have attracted growing scientific and consumer attention because of their dense profiles of polyphenols, anthocyanins, and other bioactives that underpin pronounced antioxidant activity [1, 2]. Bilberries routinely exhibit antioxidant capacities two to three times higher than those reported for cultivated blueberries, an advantage linked to the harsher alpine habitats in which bilberries mature and the resulting metabolic adaptations [3–5]. Lingonberries (*V. vitis-idaea* L.), although less ubiquitous globally, hold notable ethnobotanical significance in the diets of northern and central Europe and are increasingly attracting attention due to their robust proanthocyanidin content and dual antioxidant and antimicrobial capacities [5, 6].

Mechanistically, these phenolic constituents mediate free radical scavenging and attenuate oxidative stress, thereby providing anti‐inflammatory effects and reducing the risk of chronic diseases [7]. The natural antimicrobial constituents, such as organic acids and tannins, present in these berries contribute to their efficacy against pathogenic microbes [8]. In addition to their bioactive profiles, *Vaccinium* berries deliver substantial nutritional value, providing dietary fiber, vitamins, and essential minerals, as well as seeds enriched in polyunsaturated fatty acids (PUFAs), mainly *α*‐linolenic (omega‐3) and linoleic (omega‐6) acids [2, 9, 10]. The concentrations and bioavailability of these compounds, however, are shaped by genetic (species/cultivar), ecological, and agronomic determinants [11, 12]. Comparative analyses across berry types thus provide insights into the influence of breeding practices and environmental factors on their nutritional and functional attributes [13].

Taxonomically, *V. vitis-idaea* L. (lingonberry) and *V. myrtillus* L. (bilberries) remain predominantly collected from European forest and heathland biomes. At the same time, modern *V. corymbosum* L. (blueberry) is extensively cultivated and selected for enhanced yield and organoleptic properties [14, 15]. Concurrently, there is growing scientific and industrial impetus to characterize the biochemical uniqueness of lesser known wild taxa and genotypes, motivated by early evidence of elevated antioxidant and antimicrobial activities not always replicated in commercial cultivars [16]. Historically, lingonberries have served in phytotherapy for urogenital and gastric disorders, effects now linked to proanthocyanidins and their capacity to modulate microbial adhesion and gut health [6, 17–19].

Bilberries are acclaimed for ophthalmologic applications attributed mainly to their distinctive anthocyanin spectrum [14]. Blueberries, in turn, are widely studied for cardiometabolic benefits, with multiple clinical and preclinical studies describing modulation of glycemic response, lipid metabolism, and inflammatory pathways through polyphenol‐rich fractions [20–22]. However, systematic evaluations that integrate nutritional composition, detailed phenolic fingerprints, antioxidant potential, and antimicrobial efficacy across representative cultivars, particularly for Slovakia, remain scarce. The present work compares two lingonberry cultivars (“Linnea” and “Sanna”), three highbush blueberry cultivars (“Spartan,” “Bluecrop,” and “Chandler”), and a bilberry ecotype (“Natural”) under standardized postharvest conditions. The proximate composition, mineral and fatty acid profiles, total and individual phenolics, antioxidant capacity, and in vitro antimicrobial activity are evaluated. Moreover, the application of multivariate chemometric analysis (principal component analysis [PCA]) enables clear differentiation of berry cultivars based on their functional biochemical signatures, revealing bilberries as phenolic‐ and PUFA‐dominant, lingonberries as mineral‐ and antioxidant‐balanced, and blueberries as relatively monounsaturated fatty acid (MUFA)–rich. This integrative approach offers new evidence supporting cultivar‐specific selection of *Vacciniu*m berries for functional food and nutraceutical applications. The significance of the study will enrich the understanding of *Vaccinium* nutritional and bioactive diversity, guide cultivar selection, and support the formulation of next‐generation functional foods.

## 2. Materials and Methods

### 2.1. Plant Material

Berries from three *V. corymbosum* cultivars (“Spartan,” “Bluecrop,” and “Chandler”) and two *V. vitis-idaea* cultivars (“Linnea” and “Sanna”) were harvested at physiological maturity from a private farm in Turzovka, Slovak Republic (522 m a.s.l.). Bilberries (*V. myrtillus*, designated “Natural”) were hand‐collected from nearby coniferous forests at the same elevation for direct comparison. Immediately after harvest, the berries were placed in clean, food‐grade plastic containers, protected from direct sunlight, and transported to the laboratory within a few hours. Upon arrival, the samples were kept in the dark under refrigerated conditions (4 °C) until further processing. All fruits were dried at 40 °C for 12 h (Binder FD56, Germany), sealed in airtight containers, then milled to a fine powder (Sencor SCG1050 WH, Japan) and stored at 4 °C until analysis.

### 2.2. Chemicals and Reagents

All solvents and reference standards of analytical grade were obtained from CentralChem (Bratislava, Slovakia). Trolox, gallic acid, quercetin, caffeic acid, 2,2 ^′^‐azinobis(3‐ethylbenzothiazoline‐6‐sulfonic acid) (ABTS), DPPH, and the Folin–Ciocalteu reagent had purities of ≥ 98%.

### 2.3. Fat and Crude Fiber Content

Fat content was measured using an Ancom XT15 Fat Extractor (United States) according to the method described by Osei et al. [23]. Predried samples (1.5 g) were sealed in XT4 filter bags and subjected to petroleum ether extraction at 90 °C for 60 min. The bags were subsequently redried at 105 °C, and total crude fat content was determined from the weight difference measured before and after extraction. All proximate parameters were conducted in triplicate.

Crude fiber was quantified using the ANKOM 200 Fiber Analyzer (Ancom, United States). Predefatted samples (1 g) were sequentially hydrolyzed with 0.1 M H_2_SO_4_ and 0.1 M KOH at 100 °C for 45 min, with intermittent hot water washes and a final rinse with acetone. The resulting residues were oven‐dried at 105 °C for 2 h and ashed at 550 °C for 2 h, and the crude fiber content was calculated from the loss of organic matter, applying a standard blank correction.

### 2.4. Sample Preparation for Phytochemical Analysis

Berry powder (0.2 g) was extracted with 20 mL of 80% ethanol (*v*/*v*) under orbital shaking (2 h, 25 °C) [24]. After centrifugation at 4000 rpm for 10 min (ROTOFIX 32 A, Hettich, Germany), supernatants were used for total polyphenols, flavonoids, phenolic acids, and antioxidant capacity assays. Anthocyanins were quantified from a second extraction: 1 g of powder was sequentially washed with acidified 90% ethanol, pooled, filtered, and diluted to 50 mL. All extracts were prepared in triplicate.

### 2.5. Quantification of Antioxidant Capacity and Phenolic Compounds

#### 2.5.1. ABTS Radical Scavenging Capacity

Total antioxidant activity was evaluated using the ABTS radical cation decolorization assay optimized for berry matrices [25]. ABTS (prepared with 2.45 mM potassium persulfate, equilibrated overnight) was diluted to an absorbance of ~0.70 at 734 nm in 0.01 M PBS (pH 7.00). Samples (20 *μ*L extract) were incubated with 2 mL ABTS solution, and the absorbance was read at 6 min postaddition (Jenway 6405 UV/Vis, United Kingdom). Antioxidant capacity was standardized against Trolox (100–1000 mg/L, *R* ^2^ > 0.99), and results were presented as milligrams of Trolox equivalents per gram of dry matter.

#### 2.5.2. Phosphomolybdenum (PM)‐Reducing Capacity

Reducing power was determined using the methods previously outlined [26]. Briefly, 1 mL of sample extract was incubated with reagents (monopotassium phosphate, sulfuric acid, and ammonium heptamolybdate) at 90 °C for 120 min. Absorbance was measured at 700 nm, with Trolox used as the standard. Results were reported in milligrams of Trolox equivalents per gram of dry matter.

#### 2.5.3. Total Polyphenols, Flavonoids, and Phenolic Acids

Total polyphenols were determined via the Folin–Ciocalteu method described and modified for high‐phenolic matrices [27]. Samples (100 *μ*L) were combined with Folin–Ciocalteu reagent, sodium carbonate, and water, incubated in darkness (30 min), then read at 700 nm. Results were reported as milligrams of gallic acid equivalents per gram of dry matter.

Total polyphenol content was quantified using a modified Folin–Ciocalteu assay adapted for high‐phenolic matrices [27]. Briefly, 100 *μ*L of the sample was mixed with Folin–Ciocalteu reagent, sodium carbonate, and distilled water, then incubated in the dark for 30 min. Absorbance was measured at 700 nm, and results were expressed as milligrams of gallic acid equivalents per gram of dry matter.

Total flavonoid (TF) content was determined using a modified aluminum chloride colorimetric assay [28]. A 0.5 mL sample mixed with AlCl_3_, potassium acetate, and water was incubated for 30 min at room temperature. Absorbance was recorded at 415 nm; and the flavonoid content was expressed as milligrams of quercetin equivalents per gram of dry matter.

Total phenolic acids (TPAs) were quantified in the berry samples following the method of. Briefly, 0.5 mL of berry extract was mixed with 0.5 mL of 0.5 M HCl, 0.5 mL of Arnova reagent (10*%*NaNO_2_ + 10*%*Na_2_MoO_4_ [29]), 0.5 mL of 1 M NaOH, and 0.5 mL of distilled water. After thorough mixing, absorbance was measured at 490 nm using a Jenway 6405 UV/Vis spectrophotometer (United Kingdom). Results were expressed as milligrams of caffeic acid equivalents per gram of dry weight.

#### 2.5.4. Total Anthocyanin (TA) Content

Anthocyanin content was measured according to the method previously described and modified by [30, 31]. For pH 1.0, a sample (0.4 mL) was diluted with 0.025 M potassium chloride (3.6 mL). For pH 4.5, a sample was diluted (0.4 mL) with 0.4 M sodium acetate. The absorbance of the sample was measured at 520 and 700 nm against the blank reagent (distilled water). The concentration (milligrams per gram) of TAs was calculated according to the following formula and expressed as cyanidin‐3‐glucoside (Cy‐3‐glc) equivalent in *V. vitis-idaea* L. and delphinidin‐3‐O‐glucoside (De‐3‐glc) equivalent in *V. corymbosum* L. and *Vaccinium myrtilus* L. The analysis was carried out in triplicate. The TA content was calculated using the following formula:

Amg/g=A∗Mw∗1000ε∗L,

where *A* is the absorbance difference = (A_520_–A_700_) pH 1.0–(A_520_–A_700_) pH 4.5, *M* *w* is the molecular weight of Cy‐3‐glc = 449.2 g/mol, De‐3‐glc = 518.5 g/mol, *ε* is the extinction coefficient of Cy‐3‐glc = 2690 cm/mol, De‐3‐glc = 2900 cm/mol, and *L* is the absorption; path length in cm = 1.

#### 2.5.5. Polyphenol Composition Determination by HPLC‐DAD Method

Polyphenol composition (milligrams per gram) was determined using the separation gradient method RP‐HPLC/UV‐DAD by the Agilent 1260 Infinity high‐performance liquid chromatograph (Agilent Technologies, Waldbronn, Germany). Separation was achieved on a Purospher reverse‐phase C18 column (4 mm × 250 mm × 5 *μ*m) (Merck, KGaA, Darmstadt, Germany). The mobile consisted of (A) acetic acid in methanol (50/1000 mL) and (B) acetic acid in HPLC‐grade water (50/1000 mL). The following gradient program was employed: 0–5 min, isocratic elution (20% A and 80% B); 5–11 min, linear gradient elution (60% A and 40% B); and 11–20 min, 80% A and 20% B. The flow rate was 1 mL/min. The column oven temperature was set to 25 °C, and the samples were kept at 4 °C in the Peltier sample manager. The DAD signal was conducted at 220–400 nm with a preferred wavelength of 330 nm for quantitative purposes, with a data acquisition rate of 5 Hz [32].

### 2.6. Mineral Content Analysis

The elemental composition of the berry powder was determined following the procedure of Ivanišová et al. [33] using atomic absorption spectroscopy. The Varian model AA 240 FS, equipped with a D2 lamp background correction system and an air–acetylene flame (air flow rate: 13.5 L/min, acetylene flow rate: 2.0 L/min; Varian Ltd., Mulgrave, Australia), was employed to analyze mineral compounds. Results were compared to the multielementary GFAAS standard, CertiPUR (Merck, Germany). Samples were subjected to microwave‐assisted acid digestion using a 1:1 mixture of HNO_3_ and water (MARS X‐press, United States). Concentrations of Fe, Mn, Zn, Cu, Co, Ni, Cr, Cd, and Pb were measured by atomic absorption spectrophotometry (Varian AA 240 FS, D_2_ lamp, air–acetylene). Calibration was performed using CertiPUR multielement standards (Merck, Germany). Results were expressed as milligrams per kilogram of dry weight.

### 2.7. Fatty Acid Profile

The fatty acid profile was determined using a method previously described by Ivanišová et al. [34]. Fatty acid methyl esters were prepared from berry oil according to a standard transesterification protocol. Briefly, 0.1 g of n‐hexane‐extracted berry oil was placed in a 40 mL glass vial and saponified with 5 mL of 0.50 N methanolic sodium hydroxide at 60 °C for 3 min. After cooling to room temperature, methylation was initiated by the addition of 6 mL of a 14% boron trifluoride methanol complex. The reaction mixture was reheated at 60 °C for 3 min, cooled, and subsequently extracted with 10 mL of isooctane. Following vigorous mixing and phase separation, the upper organic layer was collected and dried over anhydrous sodium sulfate to remove residual moisture. Prior to analysis, the esterified oil was diluted at a ratio of 1:19 by combining 50 *μ*L of FAME with 950 *μ*L of n‐hexane. FAME analysis was performed using an Agilent 7890B gas chromatograph (Agilent Technologies, United States) equipped with a flame ionization detector and a CombiPAL autosampler. A 1 *μ*L aliquot of the diluted sample was injected into an HP 88 capillary column (60 m × 0.25 mm × 0.20 *μ*m; Agilent Technologies, United States). High‐purity helium, nitrogen, hydrogen, and synthetic air (Grade 5.0) were used as carrier and detector gases. Chromatographic data were acquired and processed using Agilent OpenLab ChemStation software. Quantification was based on calibration with a certified 37‐component FAME standard mixture (Supelco 37, TraceCERT CRM, Supelco, United States), and peak identification was confirmed using an Agilent 5977A mass‐selective detector operated in GC Fit mode [35]. All determinations were carried out in triplicate.

### 2.8. Antimicrobial Assays

The antimicrobial activity was evaluated using a disc‐diffusion assay [35]. Berry extracts were prepared by macerating dried berry powders (1:10) in 96% ethanol for 7 days in the dark. The extracts were then filtered, and the solvent was evaporated to dryness using a rotary evaporator (Büchi Rotavapor R‐80, Switzerland). The resulting residues were redissolved in dimethyl sulfoxide (DMSO) to obtain the final extracts used for analysis. Extract aliquots (15 *μ*L) were applied to 6 mm sterile filter paper discs and placed on nutrient agar medium plates previously inoculated with standardized microbial suspensions (10^5^ cfu/mL). The test microorganisms included *Candida glabrata* (CCM8270), *Candida albicans* (CCM8186), *Candida krusei* (CCM8271), *Candida tropicalis* (CCM8223), *Haemophilus influenzae* (CCM4454), and *Escherichia coli* (CCM3954), all obtained from the Czech Collection of Microorganisms. The plates were incubated aerobically at 37 °C for 24 h, after which the diameters of inhibition zones were measured. All experiments were carried out in triplicate.

### 2.9. Statistical Analysis

Experiments were conducted in triplicate, and data are reported as the mean ± standard deviation. Statistical significance was assessed using RStudio statistical software, which employed the Shapiro–Wilk (normality), Bartlett (variance homogeneity), and Durbin–Watson (error independence) tests. Box–Cox transformations were applied where normality or homoscedasticity assumptions were unmet (*λ*, from 2 to +2). Group comparisons employed Kruskal–Wallis (*p* < 0.05, nonparametric) or Tukey′s HSD (*p* < 0.05, parametric) as appropriate. PCA was employed to assess trait clustering and sample grouping based on the measured parameters.

## 3. Results and Discussion

### 3.1. Fat and Crude Fiber Content

**Figure 1:**
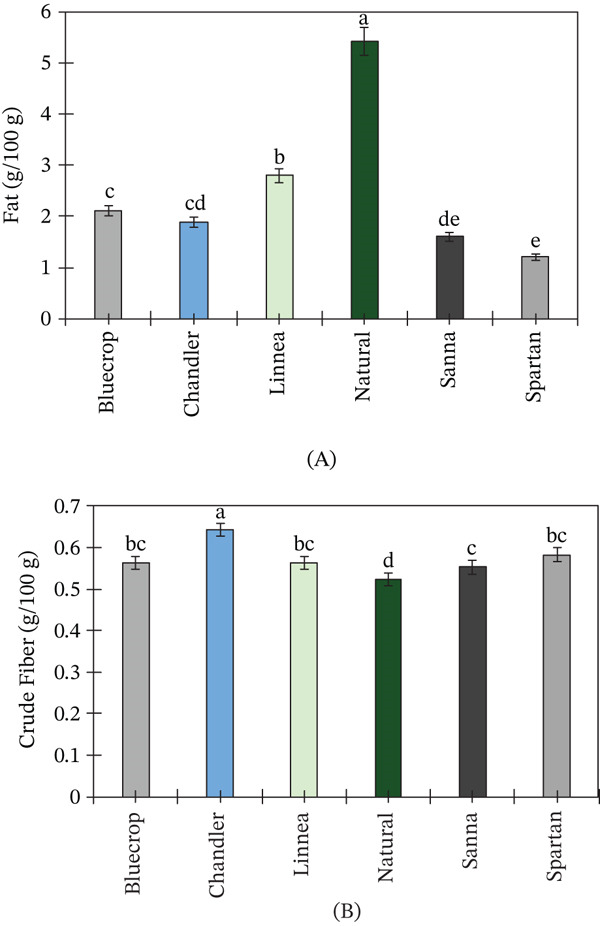
Fiber and fat composition. (A) Fat content (grams per 100 g, dry basis). (B) Crude fiber (grams per 100 g, dry basis). Bars show mean ± SD (*n* = 3). Different letters indicate significant differences among cultivars within a species at *p* < 0.05 (Tukey′s HSD). Cultivars: highbush blueberry (*V. corymbosum*) “Spartan,” “Bluecrop,” “Chandler”; lingonberry (*V. vitis-idaea*) “Linnea,” “Sanna”; bilberry (*V. myrtillus*) “Natural.”

The fat content varied significantly (*p* < 0.05) among the *Vaccinium* species and cultivars (Figure 1A). Bilberry (*V. myrtillu*s “Natural”) exhibited the highest fat content (5.42 g/100 g), followed by lingonberry “Linnea” (2.8 g/100 g). In contrast, “Spartan” highbush blueberry showed the lowest values (1.21 g/100 g), although it was not significantly different from “Sanna.” The lipid content for the wild berry (5.4 g/100 g) compares well with that of Aguilera et al. [36], who reported similar fat content in the dry fruit of *Ribes magellanicum*, although it is lower than the 8.8 g/100 g reported by Domínguez et al. [37], indicating some compositional variability between *Vaccinium* and *Ribes* genera. This observation may be due to sample disparities and variations in lipid extraction procedures, such as grinding and the type of solvent used [38]. This trend aligns with earlier reports that bilberries concentrate solids due to slower growth and environmental stress in alpine habitats [4]. The observed differences in fat content have potential nutritional implications, as berry lipids, particularly PUFAs, are associated with cardiovascular and anti‐inflammatory benefits [10].

Due to the high content of seeds and skins, fiber is an essential component of berry by‐products [39]. Crude fiber content (Figure 1B) showed that one highbush blueberry cultivar (notably “Chandler”) was significantly higher (0.64 g/100 g), whereas highbush Natural exhibited the lowest fiber content (0.52 g/100 g). A study by Probst [40] reported dietary fiber concentrations of 6.1 g/100 g in *Ribes* species such as raspberries and blackberries and 8.1 g/100 g in loganberries, generally exceeding those observed in the present *Vaccinium* samples and highlighting intergeneric differences. Similarly, Niro et al. [41] found higher fiber content in dried goji berries (11.4 g/100 g), values exceeding those observed in the present study. The inverse relationship between crude fat and fiber noted in the correlation analysis (Section 3.7) suggests cultivar‐specific allocation of structural versus lipid reserves, possibly reflecting adaptations to ecological conditions or breeding selection priorities [11].

### 3.2. Antioxidant Capacity

**Figure 2:**
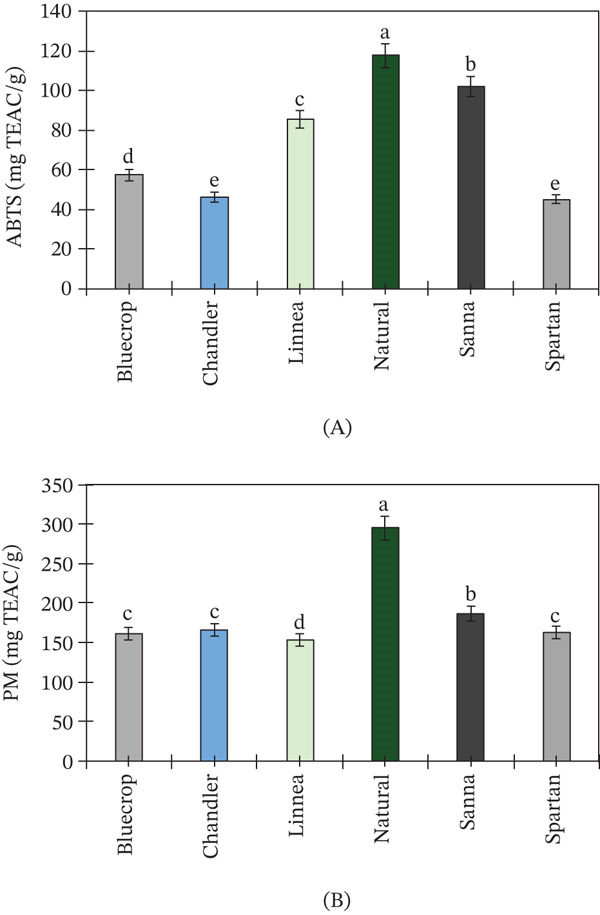
Antioxidant capacity of berry extracts. (A) ABTS radical scavenging capacity, reported as Trolox equivalents (milligrams of Trolox equivalent antioxidant capacity per gram, dry basis). (B) Total reducing capacity by the phosphomolybdenum assay (milligrams of Trolox equivalent antioxidant capacity per gram, dry basis). Bars show mean ± SD (*n* = 3). Different letters indicate significant differences among cultivars within a species at *p* < 0.05 (Tukey′s HSD).

Significant differences (*p* < 0.05) in antioxidant activity were observed among the *Vaccinium* species and cultivars, as measured by ABTS radical scavenging and PM‐reducing capacity (Figure 2). Highbush bilberry (“Natural”) recorded the highest ABTS values (117.7 mg TEAC/g), followed by lingonberry cultivars “Sanna” and “Linnea” (101.8 and 85.71 mg TEAC/g, respectively). The high antioxidant activity of these cultivars may be attributed to their elevated polyphenol, anthocyanin, and flavonoid content. Bilberries and lingonberries have abundant hydroxycinnamates, including chlorogenic acid, which further contributes to their antioxidant activity [42]. The cultivated blueberries (“Bluecrop,” “Chandler,” and “Spartan”) exhibited lower values (44.97–54.96 mg TEAC/g), indicating a comparatively reduced radical scavenging capacity. This trend supports the hypothesis that wild berries possess a higher antioxidant potential due to greater accumulation of polyphenols and anthocyanins, a consequence of adaptive responses to environmental stress [4, 5].

Prior studies confirm that bilberries tend to show greater antioxidant activity than domesticated blueberries [4, 43]. European bilberry extracts have been reported with ABTS values on the order of 200–480 *μ*mol TE/g DW, significantly higher than those of highbush blueberry [44, 45]. In this study, the ranking of antioxidant capacity mirrored the total phenolic content (TPC) across samples. This positive correlation between total phenolics and antioxidant power is well documented in berries [6, 46].

The PM‐reducing capacity results followed a similar trend, with bilberry and lingonberry outperforming all highbush blueberry cultivars. Natural had the highest PM (291.5 mg TEAC/g) and Linnea the lowest (152.9 mg TEAC/g), while no significant differences were observed among Bluecrop, Chandler, and Spartan. The stronger reducing power in bilberry and lingonberry species is consistent with their higher phenolic acid and flavonoid contents [1, 6]. The observed differences are nutritionally relevant, as higher antioxidant capacity is associated with greater potential to modulate oxidative stress and related chronic disease risks. These findings also provide a biochemical basis for cultivar selection in the development of functional foods, where antioxidant‐rich berry varieties can be prioritized for health‐promoting formulations [20, 22].

### 3.3. Phenolic Constituents

**Figure 3:**
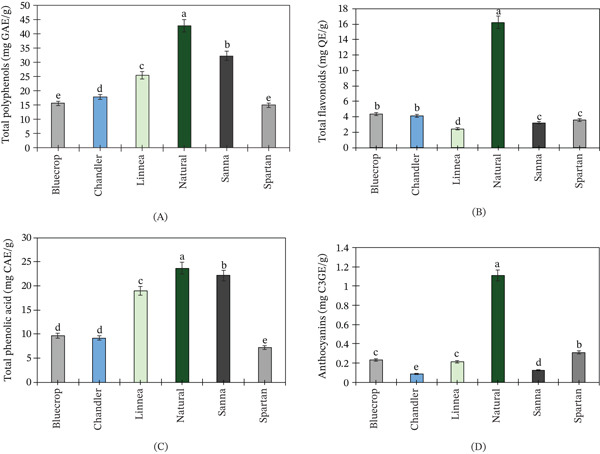
Phenolic constituents of berry extracts. (A) Total polyphenols (milligrams of gallic acid equivalents per gram). (B) Total flavonoids (milligrams of quercetin equivalents per gram). (C) Total phenolic acids (milligrams of caffeic acid equivalents per gram). (D) Total anthocyanins (milligrams of cyanidin‐3‐glucoside equivalents per gram). All values are expressed on a dry‐matter basis and plotted as mean ± SD (*n* = 3). Different letters indicate *p* < 0.05 among cultivars within a species (Tukey′s HSD).

Marked variation in TPCs, TFs, TPAs, and TAs was observed across the studied *Vaccinium* species and cultivars (Figure 3). Bilberry (“Natural”) consistently recorded the highest values for all four phenolic classes, followed by lingonberry cultivars, with blueberries showing the lowest concentrations. Statistical analysis confirmed significant differences among cultivars (*p* < 0.05). The elevated phenolic content in bilberry supports earlier findings that wild berries develop denser phenolic profiles due to increased exposure to UV light and lower temperatures in alpine environments, which induce activation of the phenylpropanoid pathway [4, 16]. A similar outcome has been reported where blueberries showed higher phenolic content compared to lingonberries [9].

Anthocyanin concentration was significantly (*p* < 0.05) higher (1.11 mg C3GE/g) in bilberry (“Natural”), consistent with its intense pigmentation, and was dominated by delphinidin and cyanidin glycosides. Moreover, lingonberries exhibited lower TAs but a distinct proanthocyanidin‐rich profile, known for antimicrobial and urinary tract health benefits [6]. The anthocyanin content of lingonberries in this study compares well with values reported earlier for samples from Poland and the United States [9, 31]. These differences may be attributed to soil characteristics, cultivation techniques, or possible variations in analytical or extraction methods used. The blueberries exhibited intermediate anthocyanin levels, primarily consisting of malvidin and petunidin derivatives. These interspecific differences suggest that cultivar choice can be tailored to achieve desired functional food outcomes, with bilberry favored for its high anthocyanin and antioxidant capacity and lingonberry for its proanthocyanidin‐driven antimicrobial properties.

HPLC profiling (Table 1) revealed that chlorogenic acid, protocatechuic acid, syringic acid, rutin, and quercetin were the dominant phenolics across species. The bilberries had particularly high protocatechuic acid, caffeic acid derivatives, and rutin, while lingonberries were enriched in quercetin and *p*‐coumaric acid. Blueberries, especially “Bluecrop” and “Spartan,” contained comparatively lower phenolic acid concentrations but notable amounts of chlorogenic acid. The phenolic profile of these berry cultivars is essential for food product development, particularly in the winter season when fresh berries are available only in limited quantities, and the market relies largely on imported or processed alternatives. Powders from these berries may be applied in food products, including tea and bakery products, as a good source of health‐promoting compounds [9]. The compositional differences in the berry cultivars in this study have functional implications, as specific phenolic acids, such as chlorogenic and caffeic acids, are linked to antioxidant, anti‐inflammatory, and glycemic‐modulating activities [1, 22]. Specifically, anthocyanin‐rich extracts from blueberries have shown the ability to improve visual function during retinal inflammation [47] and inhibit the growth of human cancer cells in vivo studies [48].

**Table 1:** Phenolic profile (milligrams per gram) of the different berry varieties.

**Sample**	**Chlorogenic acid**	**Protocatechuic acid**	***Trans*-caffeic acid**	**Syringic acid**	**Rutin**	**Vitexin**	**Ellagic acid**	***Trans*-*p*-coumaric**	***Trans*-sinapic acid**	***Trans*-ferulic acid**	**Rosmarinic acid**	**Resveratrol**	**Daidzein**	**Quercetin**	**Apigenin**	**Kaempferol**
*V. corymbosum* Bluecrop	4.33 ± 0.09^a^	0.84 ± 0.051^b^	0.017 ± 0.006^c^	0.12 ± 0.012^ab^	0.077 ± 0.006^bc^	ND	0.113 ± 0.006^a^	ND	0.023 ± 0.006^bc^	0.033 ± 0.006^b^	ND	ND	0.017 ± 0.006^a^	0.010 ± 0.000^e^	ND	0.020 ± 0.000^ab^
*V. corymbosum* Chandler	2.95 ± 0.01^b^	0.60 ± 0.015^c^	0.013 ± 0.006^cd^	0.107 ± 0.015^bc^	0.103 ± 0.0112^ab^	ND	0.017 ± 0.006	ND	0.020 ± 0.000^bc^	0.010 ± 0.000^c^	ND	ND	0.013 ± 0.006^a^	0.030 ± 0.000^c^	ND	0.010 ± 0.000^c^
*V. vitis-idaea* Linnea	0.06 ± 0.006^e^	0.13 ± 0.006^d^	ND	0.023 ± 0.006^d^	0.077 ± 0.006^bc^	ND	0.027 ± 0.006	0.020 ± 0.000^c^	0.020 ± 0.000^bc^	0.027 ± 0.006^b^	ND	ND	ND	0.047 ± 0.006^b^	ND	ND
*V. myrtillus* Natural	1.24 ± 0.02^d^	1.57 ± 0.050^a^	0.070 ± 0.006^a^	0.087 ± 0.006^c^	0.143 ± 0.006^a^	0.013 ± 0.006^a^	0.097 ± 0.006^a^	0.027 ± 0.006^b^	0.047 ± 0.006^a^	0.010 ± 0.000^c^	ND	0.087 ± 0.006^a^	ND	0.057 ± 0.006^a^	ND	0.027 ± 0.006^a^
*V. vitis-idaea* Sanna	0.12 ± 0.015^e^	0.21 ± 0.020^d^	0.047 ± 0.006^b^	0.034 ± 0.006^d^	0.043 ± 0.050^c^	ND	0.053 ± 0.006	0.037 ± 0.006^a^	0.030 ± 0.000^b^	0.050 ± 0.010^a^	ND	ND	ND	0.060 ± 0^a^	ND	ND
*V. corymbosum* Spartan	2.6 ± 0.01^c^	0.54 ± 0.016^c^	0.023 ± 0.006^c^	0.14 ± 0.010^a^	0.103 ± 0.012^ab^	ND	0.107 ± 0.015^a^	ND	0.013 ± 0.006^c^	0.027 ± 0.006^b^	ND	ND	0.020 ± 0.000^a^	0.020 ± 0^d^	ND	0.013 ± 0.006^bc^
Shapiro–Wilk	0.0018	0.2051	0.0614	0.5450	0.0024	< 0.00001	0.1436	< 0.00001	0.0400	0.3886	—	< 0.00001	0.0020	< 0.00001	—	< 0.00001
Bartlett′s test	0.003141	0.1259	< 0.00001	0.6932	0.01179	< 0.00001	0.5926	< 0.00001	< 0.00001	< 0.00001	—	< 0.00001	H	< 0.00001	—	< 0.00001
Durbin–Watson	0.5674	0.3564	0.8132	0.7822	0.6058	0.0099	0.0543	0.4242	0.8474	0.2297	—	0.9635	0.2730	0.0505	—	0.0020
Box–Cox (*λ*)	0.3838	—	0.3838	—	1.2323	—	—	—	0.0606	0.2222	—	0.2222	—	—	—	—
Tukey′s HSD test	No	Yes	No	Yes	No	No	Yes	No	No	No	—	No	No	No	—	No
Kruskal–Wallis test	Yes	No	Yes	No	Yes	Yes	No	Yes	Yes	Yes	—	Yes	Yes	Yes	—	Yes

*Note:* Results represent the mean ± standard deviation (*n* = 3), while ND represents not detected. For Shapiro–Wilk (normality test), Bartlett (test of homogeneity of variances), and Durbin–Watson (test of independence of errors), results represent the *p* value. The results of the Box–Cox transformation represent the lambda *λ* (ranging from −2.0 to +2.0) value used to adjust the data, making it more closely align with a normal distribution and/or homoscedasticity. Lowercase letters (^a,b,c,d,e^) indicate differences between the berry samples using the Kruskal–Wallis nonparametric analysis (*p* < 0.05).

### 3.4. Mineral Content, Including Risk Elements

Mineral concentrations differed markedly among species and cultivars (Table 2). Bilberry (“Natural”) showed the highest levels of Fe (65.26 ± 0.47 mg/kg), Mn (187.2 ± 1.43 mg/kg), Zn (10.63 ± 0.24 mg/kg), and Cu (4.74 ± 0.036 mg/kg). Lingonberry cultivars were also mineral‐dense, particularly “Linnea” for Mn (151.87 ± 0.14 mg/kg) and Zn (7.50 ± 0.01 mg/kg). Blueberries contained lower amounts overall; for instance, “Spartan” had Fe (30.81 ± 0.77 mg/kg) and Mn (22.80 ± 0.28 mg/kg). Letters in Table 2 indicate statistically significant differences among cultivars within each mineral. These patterns support the view that wild berries, as well as some lingonberry cultivars, can offer richer micronutrient profiles than commonly cultivated blueberries, likely due to genotype and growth‐environment effects [9].

**Table 2:** Mineral profile (milligrams per kilogram) (Fe, Mn, Zn, Cu, Co, Ni, Cr, Cd, and Pb) of different berry varieties.

Sample	Fe	Mn	Zn	Cu	Co	Ni	Cr	Cd	Pb
*V. corymbosum* Bluecrop	14.3 ± 0.37^e^	56.2 ± 0.54^d^	3.38 ± 0.04^d^	1.68 ± 0.099^c^	0.41 ± 0.01^c^	1.29 ± 0.05^bc^	ND	ND	0.29 ± 0.006^b^
*V. corymbosum* Chandler	20.0 ± 0.67^d^	23.2 ± 0.96^e^	3.18 ± 0.04^d^	1.29 ± 0.010^d^	0.47 ± 0.04^c^	1.58 ± 0.03^a^	ND	ND	0.30 ± 0.012^a^
*V. vitis-idaea* Linnea	26.0 ± 0.26^c^	151.9 ± 0.14^b^	7.50 ± 0.01^b^	2.49 ± 0.015^b^	0.19 ± 0.01^e^	0.20 ± 0.01^e^	ND	ND	ND
*V. myrtillus* Natural	65.3 ± 0.47^a^	187.2 ± 1.43^a^	10.6 ± 0.24^a^	4.74 ± 0.036^a^	0.82 ± 0.06^a^	1.33 ± 0.03^b^	ND	0.017 ± 0.006^b^	ND
*V. vitis-idaea* Sanna	18.6 ± 0.36^d^	99.1 ± 0.61^c^	6.81 ± 0.04^c^	1.40 ± 0.015^d^	0.31 ± 0.01^d^	1.22 ± 0.01^c^	ND	ND	ND
*V. corymbosum* Spartan	30.8 ± 0.77^b^	22.8 ± 0.28^e^	2.39 ± 0.01^e^	1.57 ± 0.042^c^	0.59 ± 0.03^b^	0.99 ± 0.016^d^	ND	0.210 ± 0.015^a^	ND
Shapiro–Wilk	0.3768	0.7774	0.0008	0.1057	0.4261	0.4261	—	0.0001	0.0002
Bartlett′s test	0.7392	0.1160	0.0004	0.0413	0.0923	0.0923	—	< 0.00001	< 0.00001
Durbin–Watson	0.7676	0.4515	0.6836	0.949	0.3497	0.3497	—	0.6149	0.9945
Box–Cox (*λ*)	—	—	−0.101	−1.717	—	—	—	−1.717	−1.717
Tukey′s HSD test	Yes	Yes	Yes	No	Yes	Yes	—	No	No
Kruskal–Wallis test	No	No	No	Yes	No	No	—	Yes	Yes

*Note:* Results represent the mean ± standard deviation (*n* = 3), while ND represents not detected. For Shapiro–Wilk (normality test), Bartlett (test of homogeneity of variances), and Durbin–Watson (test of independence of errors), results represent the *p* value. The results of the Box–Cox transformation represent the lambda *λ* (ranging from −2.0 to +2.0) value used to adjust the data, making it more closely align with a normal distribution and/or homoscedasticity. Lowercase letters (^a,b,c,d,e^) indicate differences between berries′ samples using the Kruskal–Wallis nonparametric analysis (*p* < 0.05) or Tukey′s HSD (honestly significant difference) parametric analysis (*p* < 0.05).

From a nutritional perspective, Fe and Zn contribute to oxygen transport and immune function, respectively; Mn is required for manganese superoxide dismutase activity, and Cu supports antioxidant enzymes and iron metabolism [49, 50]. Although berries are not primary sources of these minerals in typical diets on a fresh‐weight basis, the higher values in bilberry and lingonberry powders may be relevant for concentrated ingredients and functional formulations.

Toxic elements were essentially undetectable. Chromium was not detected in any sample. Cadmium appeared at trace levels in “Natural” (0.017 ± 0.006 mg/kg) and “Spartan” (0.21 ± 0.015 mg/kg). Lead was traced in “Bluecrop” (0.29 ± 0.006 mg/kg^1^) and “Chandler” (0.30 ± 0.012 mg/kg) and undetected in others. These values are low and consistent with general food safety benchmarks for contaminants in foods [51]. Overall, the data support the safety of the sampled berries and highlight bilberry and lingonberry as preferable choices where mineral enrichment is desired.

### 3.5. Fatty Acid Profile

The fatty acid composition varied significantly among the studied *Vaccinium* species and cultivars (Table 3). PUFAs dominated across all samples, with the highest proportions in lingonberry cultivars “Sanna” (74.60*%* ± 0.05*%*) and “Linnea” (73.33*%* ± 0.05*%*), followed by bilberry (“Natural,” 69.74*%* ± 0.11*%*). The highbush blueberry cultivars exhibited slightly lower PUFA levels, with “Chandler” (64.04*%* ± 0.57*%*) recording the lowest among all samples. Linoleic acid (C18:2 n6) and *α*‐linolenic acid (C18:3 n3) were the major PUFAs, together accounting for over 60% of total fatty acids in lingonberry and bilberry. MUFAs were highest in blueberries, particularly “Bluecrop” (23.35*%* ± 0.01*%*) and “Chandler” (22.65*%* ± 0.006*%*), mainly due to oleic acid (C18:1cis n9) content. Saturated fatty acids (SFAs) were present in lower proportions, with “Chandler” (10.48*%* ± 0.015*%*) and “Bluecrop” (9.28*%* ± 0.006*%*) showing the highest values, while lingonberry cultivars contained the least (< 3.5%). Nutritionally, the high PUFA content, especially the n3 fatty acid *α*‐linolenic acid, enhances the functional value of lingonberry and bilberry, given its established role in reducing inflammation and cardiovascular risk [52]. The favorable n6/n3 ratios observed in lingonberry (0.95–0.96) and bilberry (1.04) further underline their suitability for health‐promoting formulations. In contrast, blueberries, while higher in MUFA, exhibited less favorable n6/n3 ratios (> 1.4), which may be less optimal from a cardiovascular perspective. These findings suggest that lingonberries and bilberries are more promising candidates for incorporation into functional foods targeting lipid profile improvement.

**Table 3:** Fatty acid (percentage) composition of different berry varieties.

**Sample**	**C16**	**C16:1**	**C18**	**C18:1cis n9**	**C18:2cis n6**	**C18:3 n3**	**C:20**	**C:24**	**C22:1 n9**	**PUFA**	**MUFA**	**SFA**	**Ratio n3/n9**	**Ratio n6/n3**
*V. corymbosum* Bluecrop	6.48 ± 0.006^b^	ND	2.07 ± 0.006^b^	22.38 ± 0.610^a^	39.13 ± 0.000^c^	28.9 ± 1.74^c^	0.43 ± 0.006^a^	0.31 ± 0.006^e^	0.437 ± 0.006^d^	66.04 ± 0.02^e^	23.35 ± 0.010^a^	9.28 ± 0.006^b^	0.687 ± 0.006^c^	1.443 ± 0.012^c^
*V. corymbosum* Chandler	6.81 ± 0.006^a^	ND	2.25 ± 0.010^a^	21.69 ± 0.010^bc^	40.12 ± 0.000^b^	24.2 ± 0.01^d^	ND	0.783 ± 0.006^b^	0.947 ± 0.015^b^	64.04 ± 0.57^f^	22.65 ± 0.006^b^	10.48 ± 0.015^a^	0.607 ± 0.006^e^	1.653 ± 0.006^a^
*V. vitis-idaea* Linnea	1.32 ± 0.017^f^	ND	0.203 ± 0.006^e^	15.07 ± 0.049^d^	35.80 ± 0.030^e^	37.3 ± 0.28^a^	0.11 ± 0.010^c^	1.33 ± 0.042^a^	0.91 ± 0.030^b^	73.33 ± 0.05^b^	21.19 ± 0.031^e^	3.36 ± 0.035^e^	1.043 ± 0.006^a^	0.957 ± 0.006^e^
*V. myrtillus* Natural	4.91 ± 0.01^d^	0.173 ± 0.006^a^	1.21 ± 0.006^d^	22.03 ± 0.040^ab^	35.44 ± 0.070^f^	34.2 ± 0.11^b^	0.17 ± 0.006^b^	0.223 ± 0.006^f^	ND	69.74 ± 0.11^c^	22.34 ± 0.036^c^	6.36 ± 0.270^d^	0.967 ± 0.006^b^	1.037 ± 0.006^d^
*V. vitis-idaea* Sanna	1.57 ± 0.01^e^	0.157 ± 0.006^b^	0.227 ± 0.02^e^	15.12 ± 0.010^d^	36.44 ± 0.040^d^	38.2 ± 0.01^a^	ND	0.567 ± 0.015^c^	5.31 ± 0.050^a^	74.60 ± 0.05^a^	20.67 ± 0.045^f^	2.5 ± 0.010^f^	1.047 ± 0.006^a^	0.953 ± 0.006^e^
*V. corymbosum* Spartan	5.66 ± 0.006^c^	ND	1.71 ± 0.010^c^	21.22 ± 0.023^c^	40.83 ± 0.020^a^	25.9 ± 0.96^d^	0.41 ± 0.006^a^	0.37 ± 0.030^d^	0.763 ± 0.006^c^	67.44 ± 0.14^d^	22.13 ± 0.012^d^	8.16 ± 0.040^c^	0.647 ± 0.006^d^	1.537 ± 0.006^b^
Shapiro–Wilk	0.1582	0.0020	0.1719	< 0.00001	< 0.00001	0.0022	0.3886	0.1750	< 0.00001	0.0013	0.599	< 0.00001	0.0003	0.0246
Bartlett′s test	0.5965	< 0.00001	0.3047	< 0.00001	0.2246	< 0.00001	< 0.00001	0.0391	< 0.00001	0.0009	0.1263	< 0.00001	1.00	0.8822
Durbin–Watson	0.6220	0.6776	0.9964	0.2335	0.2335	0.8204	0.0768	0.3951	0.0098	0.3828	0.6348	0.9564	0.3708	0.3367
Box–Cox (*λ*)	—	—	—	2.00	−1.55	0.8686	0.8686	−0.3430	—	‐1.393	—	0.909	0.1818	−0.2222
Tukey′s HSD test	Yes	No	Yes	No	Yes	No	No	Yes	No	No	Yes	No	No	Yes
Kruskal–Wallis test	No	Yes	No	Yes	No	Yes	Yes	No	Yes	Yes	No	Yes	Yes	No

*Note:* Results represent the mean ± standard deviation (*n* = 3), while ND represents not detected. For Shapiro–Wilk (normality test), Bartlett (test of homogeneity of variances), and Durbin–Watson (test of independence of errors), results represent the *p* value. The results of the Box–Cox transformation represent the lambda *λ* (ranging from −2.0 to +2.0) value used to adjust the data, making it more closely align with a normal distribution and/or homoscedasticity. Lowercase letters (^a,b,c,d,e,f^) indicate differences between berry samples using the Kruskal–Wallis nonparametric analysis (*p* < 0.05).

### 3.6. Antimicrobial Activity

The antimicrobial screening revealed clear species‐ and cultivar‐specific differences in inhibition against the tested bacteria and yeasts (Table 4). Bilberry (“Natural”) exhibited the broadest and strongest inhibitory profile, producing the largest inhibition zones against *C. krusei* (5.19 ± 0.07 mm), *C. tropicalis* (5.01 ± 0.10 mm), *C. albicans* (5.79 ± 0.22 mm), *C. glabrata* (5.10 ± 0.021 mm), and *H. influenzae* (3.13 ± 0.021 mm). Lingonberry cultivar “Linnea” also demonstrated potent antifungal activity, particularly against *C. albicans* (5.59 ± 0.12 mm) and *C. tropicalis* (4.69 ± 0.15 mm). This observed antifungal activity may be attributed to the high content of PUFAs, particularly *α*‐linolenic acid (C18:3 n3), which may disrupt fungal cell membranes, increase permeability, and cause leakage of cellular contents [53, 54].

**Table 4:** Antimicrobial activity (millimeters) of different berries.

Sample	*Candida krusei* CCM8271	*Candida tropicalis* CCM8223	*Candida albicans* CCM8186	*Candida glabrata* CCM8270	*Escherichia coli* CCM3954	*Haemophylus influenza* CCM4454
*V. corymbosum* Bluecrop	3.97 ± 0.06^c^	3.64 ± 0.03^d^	2.51 ± 0.37^b^	3.13 ± 0.021^c^	0.00 ± 0.00^d^	1.66 ± 0.01^c^
*V. corymbosum* Chandler	0.00 ± 0.00^d^	1.65 ± 0.02^e^	1.09 ± 0.09^c^	0.00 ± 0.00^e^	1.07 ± 0.05^c^	2.63 ± 0.06^b^
*V. vitis-idaea* Linnea	4.24 ± 0.25^c^	4.69 ± 0.15^b^	5.59 ± 0.12^a^	4.37 ± 0.16^b^	2.12 ± 0.02^b^	0.00 ± 0.00^e^
*V. myrtillus* Natural	5.19 ± 0.07^b^	5.01 ± 0.10^a^	5.79 ± 0.22^a^	5.10 ± 0.02^a^	3.17 ± 0.07^a^	3.13 ± 0.02^a^
*V. vitis-idaea* Sanna	9.19 ± 0.16^a^	4.11 ± 0.02^c^	2.11 ± 0.02^b^	1.07 ± 0.07^d^	0.00 ± 0.00^d^	1.13 ± 0.02^d^
*V. corymbosum* Spartan	0.00 ± 0.00^d^	1.00 ± 0.01^f^	0.00 ± 0.00^d^	0.00 ± 0.00^e^	0.00 ± 0.00^d^	2.59 ± 0.28^b^
Shapiro–Wilk	0.0363	0.0188	0.0674	0.0051	0.0096	< 0.00001
Bartlett′s test	< 0.00001	0.0061	< 0.00001	< 0.00001	< 0.00001	< 0.00001
Durbin–Watson	0.0002	0.6735	0.4864	0.3478	0.8979	0.2532
Box–Cox (*λ*)	0.2222	—	—	—	—	—

In contrast, the highbush blueberry cultivars showed limited antimicrobial effects, with inhibition zones often below 3 mm and no detectable activity against several pathogens, including *C. albicans* and *E. coli* in some cases. The more vigorous activity in bilberry and lingonberry likely reflects their higher proanthocyanidin and phenolic acid contents (Section 3.3), which are known to disrupt microbial cell walls, interfere with adhesion, and inhibit enzyme activity [55].

Species‐specific patterns were evident: *E. coli* inhibition was observed only for bilberry (“Natural”) and lingonberry (“Linnea”), suggesting that specific phenolic profiles, possibly combined with organic acids, are more effective against Gram‐negative bacteria. Previous studies by Klavins et al. [56] reported potent inhibitory activity of bilberry and lingonberry extracts and lipids against bacteria, including *E. coli* and *Staphylococcus aureus*. Fatty acids can inhibit bacterial growth by inducing cell deformation, altering morphology, and ultimately disrupting the cell membrane, which leads to leakage of the cellular contents [57]. The absence of antimicrobial activity in some cultivars against *H. influenzae* and *E. coli* highlights that a high TPC alone may not guarantee broad‐spectrum antimicrobial action; instead, the qualitative composition of phenolics appears to be critical. These results support the potential of bilberry and lingonberry extracts as natural antimicrobial agents in functional foods or nutraceuticals, particularly protecting consumers against potential fungal infections [58].

### 3.7. Correlation Analysis and PCA

#### 3.7.1. Correlation Analysis

Correlation analysis (Table 5) showed strong positive associations among all antioxidant measures across the evaluated *Vaccinium* species and cultivars. ABTS, total polyphenols, TF, and phenolic acids were highly correlated (r = 0.94–0.98, *p* < 0.001), reinforcing that phenolic compounds are the primary contributors to antioxidant capacity in *Vaccinium* berries [1, 4]. These correlations also indicate that samples with higher phenolic content consistently exhibit greater radical scavenging and reducing capacity, a trend consistent with previous studies on *Vaccinium* berries, including both wild‐growing species and cultivated varieties under varying environmental conditions [16]. Micronutrients such as Fe, Zn, and Cu exhibited significant positive correlations with antioxidant parameters, suggesting synergistic interactions between polyphenols and transition metals [59]. Phenolic compounds can chelate metal ions, enhance their stability, and potentially improve redox‐related bioactivity. Lipid composition also influenced biological activity. PUFA, particularly *α*‐linolenic acid (C18:3 n3), correlated positively with inhibition of *C. krusei*, *C. tropicalis*, and *C. albicans.* In contrast, linoleic acid (C18:2 n6) was negatively correlated with antifungal activity. This agrees with previous findings that n3 PUFAs have antimicrobial properties through disruption of microbial membranes, while higher n6 PUFAs may not provide similar effects [60]. These relationships suggest that cultivars combining high‐phenolic content with favorable PUFA profiles may offer both antioxidant and antimicrobial advantages.

**Table 5:** Linear correlations among berry varieties using proximate, bioactive compounds, antioxidant capacity, fatty acids, and microbials.

Correlations	PM	ABTS	Polyphenols	Flavonoids	Phenolic acids	Fat	Crude fiber	Fe	Zn	Cu	Chlorogenic acid	Proto catechuic acid	Syringic acid	C18:2cis n6	C18:3 n3	C22:1 n9	UFA	*Candida krusei* CCM8271	*Candida tropicalis* CCM8223	*Candida albicans* CCM8186
ABTS	**0.729** ^*^																			
Polyphenols	0.853^**^	0.962^***^																		
Flavonoids	**0.971** ^***^	**0.601**	**0.739** ^*^																	
Phenolic acids	0.635	0.984^***^	0.939^***^	0.493																
Fat	**0.873** ^**^	**0.714** ^*^	**0.787** ^**^	**0.908**	**0.643**															
Crude fiber	−0.582	−0.771^**^	−0.679^*^	−0.525	−0.698	−0.576														
Fe	**0.907** ^***^	**0.596**	**0.732** ^*^	**0.921** ^***^	**0.508**	**0.866** ^**^	**−0.529**													
Zn	0.767^**^	0.96^***^	0.957^***^	0.686^*^	0.948^***^	0.841^**^	−0.717^*^	0.712^*^												
Cu	**0.88** ^**^	**0.714** ^*^	**0.785** ^**^	**0.909** ^***^	**0.634** ^*^	**0.972** ^***^	**−0.660**	**0.931** ^***^	**0.839** ^**^											
Chlorogenic acid	−0.24	−0.726^*^	−0.667^*^	−0.067	−0.81^**^	−0.26	0.406	−0.276	−0.701^*^	−0.306										
Protocatechuic acid	**0.824** ^**^	**0.294**	**0.441**	**0.92** ^***^	**0.156**	**0.759** ^**^	**−0.361**	**0.761** ^**^	**0.379**	**0.744** ^*^	**0.328**									
Syringic acid	−0.039	−0.662^*^	−0.536	0.113	−0.768^**^	−0.181	0.307	0.025	−0.622	−0.144	0.869^**^	0.447								
C18:2cis n6	**−0.541**	**−0.944** ^***^	**−0.862** ^**^	**−0.437**	**−0.964** ^***^	**−0.674** ^*^	**0.714** ^*^	**−0.457**	**−0.945** ^***^	**−0.657** ^*^	**0.771** ^**^	**−0.121**	**0.800** ^**^							
C18:3 n3	0.280	0.851^**^	0.701^*^	0.132	0.89^**^	0.36	−0.700^*^	0.191	0.773^**^	0.377	−0.829^**^	−0.186	−0.875^**^	−0.915^***^						
C22:1 n9	**−0.229**	**0.305**	**0.183**	**−0.420**	**0.408**	**−0.317**	**−0.11**	**−0.371**	**0.141**	**−0.338**	**−0.595**	**−0.630**	**−0.666** ^*^	**−0.335**	**0.564**					
PUFA	0.133	0.736^*^	0.586	−0.045	0.794^**^	0.146	−0.604	0.106	0.634	0.207	−0.901^***^	−0.382	−0.857^**^	−0.784^**^	0.94^***^	0.675				
*Candida krusei* CCM8271	**0.321**	**0.807** ^**^	**0.666** ^*^	**0.151**	**0.816** ^**^	**0.245**	**−0.681** ^*^	**0.055**	**0.635**	**0.223**	**−0.594**	**−0.073**	**−0.696** ^*^	**−0.775** ^**^	**0.874** ^**^	**0.596**	**0.797** ^**^			
*Candida tropicalis* CCM8223	0.491	0.869^**^	0.751^**^	0.430	0.861^**^	0.678^*^	−0.754^**^	0.359	0.861^**^	0.632	−0.544	0.209	−0.682^*^	−0.944^***^	0.853^**^	0.223	0.655	0.783^**^		
*Candida albican*s CCM8186	**0.530**	**0.762** ^**^	**0.705** ^*^	**0.536**	**0.758** ^**^	**0.825** ^**^	**−0.630**	**0.551**	**0.873** ^**^	**0.793** ^**^	**−0.513**	**0.314**	**−0.580**	**−0.882** ^**^	**0.68** ^*^	**−0.027**	**0.488**	**0.455**	**0.896** ^**^	
*Candida glabrata* CCM8270	0.523	0.671^*^	0.595	0.570	0.623	0.819^**^	−0.713^**^	0.541	0.772^**^	0.803^**^	−0.289	0.441	−0.375	−0.773^**^	0.583	−0.183	0.361	0.409	0.871^**^	0.948^***^

*Note:* Values in bold indicate very strong (*p* value < 0.001), strong (*p* value < 0.01), or moderate (*p* value < 0.05) correlation between dependent variables.

^*^ *p* value < 0.05. ^**^ *p* value < 0.01. ^***^ *p* value < 0.001.

#### 3.7.2. PCA

**Figure 4:**
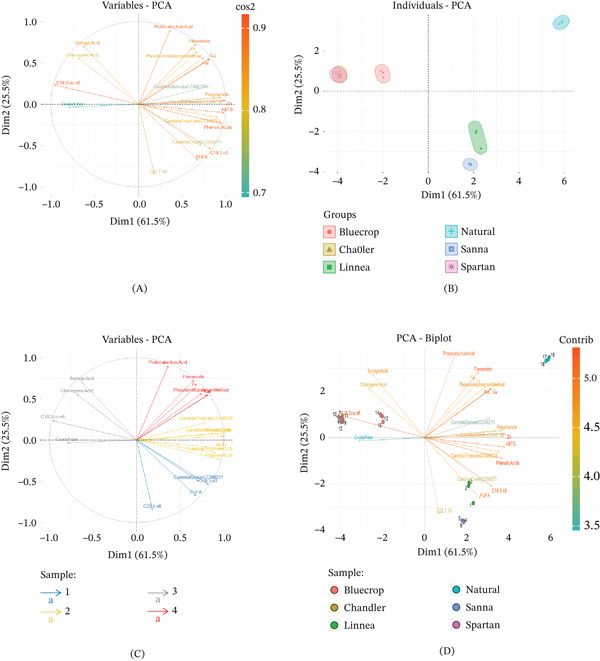
Principal component analysis (PCA) showing (A, C) two‐factor maps and (B, D) score projections by berries varieties (Linnea, Sanna, Chandler, Spartan, Bluecrop, and Natural) for proximate results (crude, protein, and fat,), bioactive compounds (chlorogenic acid, proto catechuic acid, syringic acid, total phenolic acid, total polyphenols, and total flavonoids), phosphomolybdenum method and ABTS antioxidant capacity, some important minerals (Fe, Zn, and Cu), some important fatty acids (linoleic: C18:2cis n6, linolenic: C18:3 n3, erucic: C22:1 n9, and polyunsaturated fatty acid [PUFA]), and some *Candida* species (*C. krusei* CCM8271, *C. tropicalis* CCM8223, and *C. albicans* CCM8186. (A) Variable′s graph showing the Principal Component 1 (PC 1, Dim1 = 61.5*%*) and PC 2 (Dim2 = 25.5*%*) variability impact (cos2). (B) Individual′s graph showing the two PCAs (PC 1 and PC 2) for each berry variety linking. (C) Variable′s graph showing the PC 1 (Dim1 = 61.5*%*) and PC 2 (Dim2 = 25.5*%*) clustering. (D) Biplot graph showing the PCA by berry varieties and their degree of contribution.

PCA reduced the 21 measured variables to three principal components, explaining 92.1% of the total variance (PC 1 = 61.5*%*, PC 2 = 25.5*%*, and PC 3 = 5.58*%*) (Table 6 and Figure 4). PC 1 (Figure 4A) was strongly associated with antioxidant traits (ABTS, PM, polyphenols, flavonoids, and phenolic acids) and PUFAs, as reported in earlier studies [4, 61–64], effectively separating bilberry (“Natural”) and lingonberry cultivars from all the highbush blueberry cultivars. This indicates that antioxidant‐rich, PUFA‐dense cultivars form a distinct biochemical group, consistent with their superior functional potential. Moreover, the coalignment of phenolic‐rich profiles with *Candida* inhibition outcomes is consistent with evidence that phenolic acids and flavonoids can inhibit *Candida* spp. through multifactorial mechanisms (e.g., membrane disruption, oxidative/mitochondrial effects, and virulence modulation) [64–66].

**Table 6:** PC pattern for the first (PC 1), second (PC 2), and third (PC 3) components (dimensions), eigenvalues, and variances explained among compounds for the berry varieties.

Features	PC 1	PC 2	PC 3
PM	0.7421	0.5643	0.3452
ABTS	0.9769	−0.1022	0.1500
Polyphenols	0.9429	0.0794	0.2981
Flavonoids	0.6550	0.7245	0.1987
Phenolic acids	0.9558	−0.2302	0.1467
Fat	0.8110	0.5439	−0.0987
Crude fiber	−0.7953	−0.0351	0.0762
Fe	0.6664	0.6305	0.2499
Zn	0.9891	0.0454	0.0390
Cu	0.8144	0.5483	−0.0474
Chlorogenic acid	−0.6842	0.5538	−0.2375
Protocatechuic acid	0.3610	0.8976	0.0822
Syringic acid	−0.6282	0.7039	0.0785
C18:2cis n6	−0.9601	0.2352	0.1160
C18:3 n3	0.8226	−0.5398	−0.0749
C22:1 n9	0.1813	−0.8479	0.2989
PUFA	0.6798	−0.6755	0.0823
*Candida krusei* CCM8271	0.7116	−0.4954	0.0874
*Candida tropicalis* CCM8223	0.9048	−0.1530	−0.3353
*Candida albican*s CCM8186	0.8707	0.0773	−0.4336
*Candida glabrata* CCM8270	0.7984	0.2188	−0.5527
Eigenvalues	12.91	5.36	1.17
Variance (%)	61.5	25.5	5.58

Projections of the individual scores on the 2D map of quality features for the two principal components, PC 1 versus PC 2 (Figure 4B,D), show that *C. glabrata* (CCM8270) is closely associated with polyphenols, flavonoids, and phenolic acids, indicating a possible relationship with antioxidant‐rich environments; *C. albicans* (CCM8186) and *C. tropicalis* (CCM8323) are linked to ABTS, Zn, and phenolic acids, reinforcing their connection with antioxidant activity; and *C. krusei* (CCM8271) correlates strongly with PUFA and C18:3 n3, suggesting an affinity for PUFAs. PC 3 accounted for antimicrobial activity variation, primarily linked to inhibition of *Candida* spp. The score plot (Figure 4B) revealed apparent clustering: Bilberry (“Natural”) was positioned far from other cultivars due to its exceptional antioxidant and PUFA profile. Lingonberries clustered closely with balanced phenolic and mineral traits, and blueberries grouped with lower antioxidant and antimicrobial loadings. These findings corroborate the correlation analysis, confirming that phenolic‐ and PUFA‐rich cultivars tend to have enhanced antifungal potential [64, 65]. Such multivariate differentiation provides a valuable basis for cultivar selection in functional food applications, allowing targeted use of bilberry for maximum antioxidant benefit and lingonberry for combined antioxidant and mineral enrichment.

PC 2 was driven by crude fiber and minerals (Fe, Zn, and Cu), with inverse loadings for certain fatty acids, distinguishing lingonberry cultivars (“Linnea” and “Sanna”) from blueberries due to their high mineral–fiber combination, as opposed to the higher lipid fractions in blueberries (Figure 4C). This pattern suggests that cultivar differentiation is dependent on which phenolic acids/flavonoid pools predominate, alongside a contrasting contribution from a specific fatty acid marker, a behavior widely reported in *Vaccinium* chemometrics, where PCA separates the berry samples by phenolic composition rather than TPC [4, 62]. Ultimately, retention of the three PCs is justified both by the high cumulative variance captured (92.1%) and by the eigenvalue > 1 criterion; nevertheless, best practice recommends corroborating retention with a scree test and parallel analysis, particularly when the variable‐to‐sample ratio is high, and component stability may be sample‐size sensitive [67, 68].

## 4. Conclusions

The comparative analysis of bilberry, lingonberry, and highbush blueberry cultivars demonstrated apparent species‐specific differences in nutritional composition, phenolic profiles, antioxidant activity, mineral content, and antimicrobial potential. All berries were harvested at physiological maturity using manual collection methods, ensuring consistency in ripeness and minimizing mechanical damage, which is critical for preserving their biochemical integrity. Based on the results of this study, berry cultivars cultivated in the selected regions of Slovakia hold substantial nutritional and functional potential. Overall, the findings not only validate the study objectives of linking biochemical composition with functional properties but also offer practical guidance for the berry industry and food technologists in selecting *Vaccinium* species for specific health‐oriented food products and nutraceutical development. Future research should investigate how processing, formulation, and storage influence the stability, bioavailability, and bioactivity of phenolics and PUFAs, alongside validating their effects in cell lines and in vivo models. Additionally, further work should assess functional performance in real food systems, consumer acceptance, and sustainable cultivar utilization.

## Author Contributions

Emmanuel Duah Osei: conceptualization, formal analysis, investigation, visualization, writing—original draft, and writing—review and editing. Anthony Amotoe‐Bondzie: visualization and writing—review and editing. Alfred Elikem Kwami Afedzi: formal analysis, investigation, visualization, and writing—original draft. Eva Ivanišová: conceptualization, data curation, formal analysis, funding acquisition, investigation, methodology, project administration, resources, supervision, validation, and writing—review and editing. Christian R. Encina‐Zelada: conceptualization, data curation, funding acquisition, project administration, resources, software, supervision, validation, visualization, and writing—review and editing. Ľuboš Harangozo: formal analysis and methodology. Július Árvay: formal analysis and methodology. Dominika Ďurkáčová: formal analysis and methodology. Branislav Gálik: methodology, validation, and writing—review and editing. Miroslava Kačániová: formal analysis and methodology. Newlove Akowuah Afoakwah: conceptualization, supervision, validation, and writing—review and editing.

## Funding

This study was funded by the project APVV‐24‐0190 development of innovative, health‐promoting beverages based on fruits, medicinal plants, and snacks.

## Conflicts of Interest

The authors declare no conflicts of interest.

## Data Availability

All data supporting this article have been included as part of the manuscript.
